# From reviews to real-time: dynamic evidence in dentistry

**DOI:** 10.1038/s41432-026-01206-2

**Published:** 2026-02-24

**Authors:** A. V. Gavrilova, C. Galli

**Affiliations:** 1 Department of Biosciences, University of Milan, Milan, Italy; 2 Department of Medicine and Surgery, Histology and Embryology Lab, University of Parma, Parma, Italy

**Keywords:** Dental public health, Oral diseases

## Abstract

**Background:**

The exponential growth of biomedical literature—over a million new PubMed entries each year—has outpaced traditional evidence-synthesis methods. Systematic reviews, long the cornerstone of evidence-based dentistry, are resource-intensive and often outdated within a few years, widening the gap between current research and clinical practice.

**Methods:**

We outline Retrieval-Augmented Generation (RAG) as a methodology for dynamic evidence reviews. RAG strengthens Large Language Models (LLMs) by combining their generative capacity with real-time retrieval from a continuously updated, curated knowledge base. This design grounds every answer in verifiable sources and mitigates the factual errors and hallucinations seen in standalone LLMs.

**Results/Implications:**

RAG enables on-demand dynamic synthesis of the latest evidence, allowing clinicians and researchers to ask complex, natural-language questions and receive concise, fully cited answers. For dental clinicians, this approach enables rapid, citation-linked answers to practice-relevant questions—such as material selection, healing outcomes, or procedural comparisons—without relying on outdated narrative summaries. We describe three complementary integration pathways—RAG on pre-retrieved article pools, public living review portals, and machine-actionable journal publications—each with distinct requirements and benefits. Looking forward, emerging *agentic AI* systems, capable of planning multi-step searches and iterative updates, may further enhance these capabilities. Although this framework is conceptually grounded and supported by emerging methodological evidence, prospective empirical validation, benchmarking against existing review approaches, and real-world deployment studies will be required to fully assess its performance, reliability, and impact on clinical decision-making.

**Conclusion:**

RAG offers a scalable, transparent alternative to static systematic reviews and can shorten the research-to-practice timeline. By automating retrieval and initial synthesis while keeping human critical appraisal and ethical judgment central, it points toward an era of augmented rather than automated intelligence in evidence-based dentistry.

Practice points

- Traditional systematic reviews, while valuable, are static snapshots of evidence and may be outdated, especially in rapidly evolving areas of dentistry, while Retrieval-Augmented Generation (RAG) can provide dynamic evidence summaries.
- Biomedical RAG systems can search the latest literature in real-time to answer specific clinical questions and have the potential to increase trustworthiness by grounding every claim in a verifiable, cited scientific source.
- The role of the scholar is evolving from primarily searching for evidence to critically appraising and applying the synthesized evidence provided by these advanced AI tools—which may soon analyze not just text but also tables, figures, and images—to individual patient care.

## Introduction: the crisis of evidence synthesis in the era of big literature

Evidence-based practice underpins modern dentistry^1,2^, but the evidence base itself is growing faster than human capacity to synthesize it^3^. Repositories such as PubMed now index over one million new papers each year, contributing to a global biomedical knowledge base that doubles in size approximately every 13 to 15 years^4^, making it ever harder for scholars to extract actionable insights^5^.

This information overload is compounded by a *publish or perish* academic culture that incentivizes publication volume, sometimes at the expense of quality^6,7^. The proliferation of narrative reviews, which require no new data, often serves more to advance careers than to genuinely advance knowledge, further cluttering the literature^8,9^. This creates a difficult environment for evidence synthesis, where the signal of high-quality research is increasingly difficult to distinguish from the noise^10^.

The traditional gold standard to identify published evidence in clinical fields, the systematic review, might prove hardly sustainable in the long run. A single review conducted by a team of five researchers can take up to 67 weeks to complete^11^, and the value of this significant time and effort investment diminishes rapidly; research indicates that in some areas over half of the reviews are outdated within 5.5 years^12^. In fast-moving fields of dentistry, such as implantology or materials science, this window of relevance may be even shorter. The result is a systemic crisis of utility: our primary tool for making sense of research is too slow and expensive to keep pace with the science it is meant to synthesize. This lag erodes clinicians’ trust in the evidence base, as they are forced to rely on summaries they know are likely incomplete or superseded, threatening the integrity of the entire evidence-based model.

At the heart of this methodological pain point is an over-reliance on keyword-based searching^13^. For decades, researchers have crafted intricate Boolean search strings, yet this approach is fragile. It lacks semantic understanding and is easily defeated by the linguistic complexity of scientific literature, where a single concept may have numerous lexical variants and closely related concepts^14^. A single missed keyword can exclude crucial studies from the results. Furthermore, the evidence is fragmented across multiple databases like MEDLINE, Embase, and the Cochrane Library^15^. Achieving comprehensive coverage often requires querying several separate databases^16^, yet studies show that even with this effort, approximately 60% of reviews fail to retrieve substantial amounts of relevant articles^17^. This patch-and-mend approach risks to be no longer tenable, necessitating a paradigm shift toward a more intelligent, dynamic, and scalable methodology for evidence synthesis^18^.

Recent advances in artificial intelligence suggest a way forward. Among them, Large Language Models (LLMs) have emerged as powerful tools for representing meaning beyond simple keyword matches^19^. These developments have opened the possibility of moving beyond static evidence summaries toward what we refer to here as *dynamic reviews*: evidence syntheses that are generated on demand from a continuously updated corpus rather than fixed at the time of publication.

## Generative AI in scientific inquiry: promise and peril

LLMs are trained to predict the next token in a sequence, and through this process they acquire deep statistical models of language and world knowledge^20^. Built on transformer architectures with attention mechanisms that capture relationships across long passages^21^, they represent complex concepts and produce coherent, contextually informed scientific text^22^. Their strength lies in rapidly distilling complex information. An LLM can summarize the methods and results of a lengthy randomized controlled trial or extract key data from multiple articles^23^. Current high-end models are already capable of sorely needed tasks in literature analysis, e.g. sorting papers of interest from PubMed-retrieved abstracts^24,25^. One might therefore be tempted to let them compose entire literature reviews or to query them directly for needed facts^26^ and their use is actually expanding among scholars and researchers^27^. LLMs may work only on their pre-trained knowledge or be linked to external resources such as web search APIs or curated retrieval systems. In the first case, their knowledge is fixed at the model’s training cutoff; in the second, they can access and summarize new documents in real time^28^. This distinction is crucial when assessing the potential of LLMs in evidence synthesis.

In practice, LLMs can potentially support evidence synthesis in at least three main ways^29,30^: (i) generating large bibliographic corpora^31^, (ii) identifying and selecting relevant full-text articles^32,33^, and (iii) summarizing and integrating the evidence once the pool is finalized^34^. They hold great promise for all these tasks but using them alone in evidence-based dentistry is risky^35^. Their knowledge is static, blind to research, guidelines, or approvals appearing after their last training update^36^—a serious flaw when currency is critical. They are also prone to hallucination: because they generate text from statistical patterns rather than genuine understanding, they may invent data, fabricate citations, or describe non-existent clinical trials^37,38^, even when connected to the web. A third limitation is opacity: LLMs tend to display a black box behavior, quite unable to explain their reasoning or reliably trace claims to source documents, even when specifically prompted^39,40^. Such weaknesses undermine transparency, verifiability, and reproducibility. The real danger is not merely error but the authority with which error is expressed: a false claim can read as convincingly as a genuine finding, risking misuse by busy users untrained in AI literacy.

## Current use of LLMs in medical and dental evidence synthesis

Building on these general capabilities and limitations, recent years have seen a rapid expansion of empirical research examining how LLMs are being incorporated into real-world evidence-synthesis workflows in medicine and, to a more limited extent, dentistry^41,42^. Rather than being deployed as autonomous review authors, LLMs have predominantly been used to augment specific, well-defined tasks within conventional systematic review pipelines, including reviews addressing clinically relevant dental questions. Empirical studies demonstrate that LLMs can assist with title and abstract screening, full-text eligibility assessment, data extraction, and preliminary summarization, often achieving substantial reductions in reviewer workload while maintaining acceptable sensitivity and specificity when used with human oversight^43,44^.

Several evaluations have shown that LLM-assisted screening can markedly reduce the time required to identify relevant studies, with false-negative rates comparable to traditional dual-reviewer approaches after iterative prompt refinement^26,43^. Other studies have extended this approach to multi-stage pipelines, in which LLMs support search strategy formulation, study selection, and structured data extraction^41,44^. Importantly, these applications remain largely task-specific: LLMs are deployed as assistive tools operating on predefined, static datasets and are embedded within otherwise conventional systematic review processes.

In dentistry, early applications of LLMs have followed a similar pattern^26,45^. Initial studies have explored their use in screening dental literature, including in imaging- and materials-focused reviews, highlighting the potential for workload reduction in domains characterized by heterogeneous terminology and rapidly evolving techniques. However, as in the broader medical literature, these approaches do not fundamentally alter the nature of the review output, which remains a fixed narrative or quantitative synthesis produced at a specific point in time.

These limitations point to the need for a more integrated approach, in which retrieval and synthesis are tightly coupled, continuously updated evidence can be accessed on demand, and generated conclusions are explicitly grounded in verifiable sources. Addressing these challenges requires moving beyond task-level automation toward methodologies that reconceptualize how evidence synthesis itself is performed—by tightly coupling retrieval, synthesis, and provenance within a unified framework.

## A grounded methodology: the RAG-powered dynamic review engine

These limitations highlight a central requirement for any AI-assisted review, particularly in clinical fields such as dentistry: every claim must be traceable to verifiable sources^46^. Simple web-connected LLMs help with knowledge cut-off but still cannot ensure systematic retrieval or citation fidelity. To meet these demands, Retrieval-Augmented Generation (RAG) combines the generative power of LLMs with a controlled, continuously updated literature corpus, ensuring that answers are grounded in identified, citable documents^47^, creating dynamic reviews. The RAG workflow unfolds in four key steps.

### Step 1: Corpus ingestion and processing (“building the library”)

A high-quality corpus—such as PubMed, the Cochrane Library, specialty journals, or guidelines—is curated and divided into coherent, self-contained ‘chunks’ (e.g., individual paragraphs or *Methods* sections) for precise indexing^48^.

### Step 2: Embedding and indexing (“creating the index cards”)

Every chunk is transformed into a numerical embedding, a high-dimensional vector that encodes the semantic meaning of the text^49^. This enables meaning-based retrieval—for example, a query on managing tooth decay can find material discussing caries prevention even without matching terms. These vectors are stored in a specialized *vector database*, which allows rapid mathematical comparison of their positions in this semantic space^50^.

### Step 3: Hybrid retrieval (“finding the right pages”)

When a user submits a question, the system converts the query itself into an embedding and calculates its distance (or similarity) to the stored vectors—typically using cosine similarity or another nearest-neighbor metric^51^. The closest matches are those whose meaning most closely aligns with the query. In parallel, a sparse keyword search can run to guarantee that critical exact terms (e.g., drug names or trial IDs) are not missed^52^. The two result sets are merged and ranked to produce the most relevant passages.

### Step 4: Prompt augmentation and generation (“writing the answer”)

**Fig. 1:**
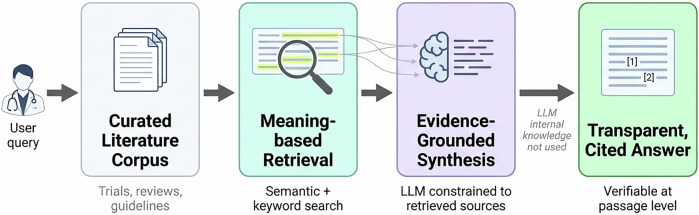
Retrieval-Augmented Generation (RAG) workflow for dynamic evidence synthesis. A user query is addressed by interrogating a curated literature corpus containing clinical trials, reviews, and guidelines. Relevant evidence is retrieved using meaning-based search that combines semantic similarity with keyword matching. The retrieved passages are then provided to a large language model, which is constrained to synthesize an answer exclusively from these sources rather than from its internal training data. The final output is a transparent, citation-linked response that can be verified at the passage level.

The highest-ranked passages are combined with the user’s question into an enriched prompt that is fed to the LLM^53–55^. The model is instructed to compose a fluent answer using only these retrieved texts and to cite each source, so every statement can be traced back to verifiable evidence (Fig. 1).

Across these levels of use, two requirements recur: systematic retrieval and verifiable provenance. Plain web-connected LLMs may reduce the knowledge-cutoff problem but cannot ensure recall, transparency, or faithful citation. RAG directly meets these needs by constraining generation to a curated, auditable corpus and exposing the exact passages supporting every claim. This approach thus transforms the LLM from an opaque text generator into a transparent scientific instrument. Each statement can be checked, verified, or refuted, making RAG compatible with the rigorous standards of evidence-based dentistry. Advanced systems can even refine searches iteratively: if an initial answer seems incomplete, the system can generate a follow-up query to deepen retrieval, mimicking how a human researcher narrows a topic after an initial broad search.

Before turning to how RAG-based reviews contrast with traditional methods, it is useful to clarify where RAG contributes within the evidence-synthesis workflow. RAG does not replace the initial step of assembling a corpus through established search methods. Rather, once this pool has been created—whether manually, through automation tools, or with LLM-assisted querying—RAG enhances the internal stages of evidence handling: it improves retrieval within the corpus through semantic and hybrid search, supports screening and data extraction by surfacing conceptually relevant passages, and enables the generation of grounded, citation-linked summaries. In other words, RAG augments the identification, extraction, and synthesis phases of evidence review, while remaining dependent on conventional search strategies to define the literature corpus itself.

While the workflow above explains the mechanics of RAG, its practical value becomes clearer when illustrated through a realistic use case that encompasses both system setup and user interaction. Imagine a research group or journal building a dynamic evidence corpus for topics related to ridge preservation. The process begins with the ingestion of a curated set of sources—randomized trials, observational studies, systematic reviews, clinical guidelines, and key journals in implant dentistry. These documents are automatically segmented into coherent text units and converted into embeddings that capture their semantic content. The resulting indexed corpus is continuously updated as new studies are published, so the system remains aligned with the current literature.

Now consider the end user—e.g. a clinician preparing a ridge-preservation case who wonders whether adjunctive hyaluronic acid improves early soft-tissue healing compared with standard care. In the traditional model, answering this question requires navigating multiple databases, identifying relevant trials, reading full texts, and manually synthesizing findings—a process measured in days or weeks. In the RAG-based system, the clinician simply poses the question in natural language.

The system interprets the query semantically and searches the curated corpus, retrieving passages from randomized controlled trials, histologic studies, and prior reviews that report wound-healing indices, collagen maturation, or epithelialization in HA-treated sockets. Because the underlying retrieval is semantic rather than keyword-dependent, the system surfaces studies even when they use diverse terminology—“*mucosal repair*”, “*early healing outcomes*”, “*ECM hydration*”—but address the same biological construct. The clinician sees not only the synthesized answer but also the exact text fragments on which it is based, each linked to its location in the source document for rapid verification.

A grounded response might read: “*Across randomized clinical studies, adjunctive hyaluronic acid is associated with improved early soft-tissue healing during the first 2–4 weeks following ridge preservation, including higher wound-healing scores and accelerated collagen organization [Ref1; Ref2]. Long-term soft-tissue thickness outcomes, however, remain inconsistent across trials [Ref3]*.” Each citation expands to show the retrieved evidence that supports the statement.

If the clinician then wishes to narrow the question—for example, to “*only RCTs with at least 3-month follow-up*”—the system automatically performs a new retrieval cycle using the refined criteria. Every generated claim remains anchored in retrieved evidence, and each step is auditable. This example illustrates how a RAG-powered system, once established, transforms literature synthesis from a static retrospective activity into an interactive, real-time dialogue with a continuously updated evidence base. Having illustrated how a RAG-based system operates in practice, it is important to situate this approach within the broader landscape of continuously updated evidence synthesis methods. This distinction matters clinically because it determines whether dentists interact with a fixed document or with an evidence base that can be queried and updated in real time.

## Dynamic reviews, living reviews, and human-curated knowledge bases

The concept of a living review has emerged in response to the increasing pace of biomedical research, particularly in fields where evidence evolves rapidly^56^. In its strict methodological definition, a living systematic review is a human-led process that follows a predefined protocol and is updated at regular intervals or when new evidence becomes available^57^. Despite being described as *living*, such reviews remain document-centric: updates occur episodically, result in discrete versions, and require the repeated execution of conventional systematic review steps, including database searching, screening, data extraction, and synthesis^58^. While this approach improves timeliness relative to static reviews, it remains resource-intensive, difficult to sustain, and limited in scalability, as documented in multiple evaluations of living review initiatives^59^.

Closely related but methodologically distinct are continuously updated, expert-curated clinical knowledge resources such as *UpToDate*^60^. These platforms represent an important precedent for dynamic access to medical knowledge and have demonstrated substantial clinical utility. However, they are not designed as systematic reviews and do not aim to provide exhaustive or reproducible evidence synthesis. Their content is selectively curated by experts, updated according to editorial judgment, and largely opaque with respect to search strategies, inclusion criteria, and completeness of evidence retrieval. As such, they prioritize usability and clinical relevance over transparency and methodological auditability.

RAG-based systems differ fundamentally from both living systematic reviews and human-curated knowledge bases. Rather than updating a canonical document or relying on editorial synthesis, a RAG-powered *dynamic review* generates evidence syntheses *on demand*, at the time of the user’s query. The review is therefore not a static or periodically updated artifact, but a computed object instantiated dynamically from a continuously updated corpus. This shift from document revision to query-time synthesis has important methodological implications. First, it decouples evidence synthesis from fixed questions, allowing users to interrogate the same corpus with multiple, evolving queries without rewriting the review. Second, it enables continuous incorporation of new evidence without requiring scheduled human re-analysis. Third, it provides passage-level provenance, exposing the specific text fragments supporting each generated claim and thereby preserving transparency and verifiability.

Importantly, RAG systems do not replace human judgment or established review methodologies. Instead, they function as computational instruments that augment evidence handling by improving internal retrieval, accelerating screening and extraction, and generating constrained, citation-anchored summaries. In contrast to living reviews, which aim to keep a document up to date, dynamic RAG-based reviews aim to keep the *evidence interaction* up to date. This distinction justifies the use of the term “dynamic review” to describe RAG-enabled synthesis systems and highlights their role as a complementary—rather than competing—paradigm within evidence-based dentistry. At present, RAG-powered dynamic reviews should be understood as a methodological framework rather than a validated replacement for systematic reviews; their value lies in restructuring evidence access and synthesis, not yet in altering clinical conclusions. The next question, however, is how such systems can be integrated into existing evidence-synthesis workflows.

## From static reviews to dynamic RAG: integration pathways

**Fig. 2:**
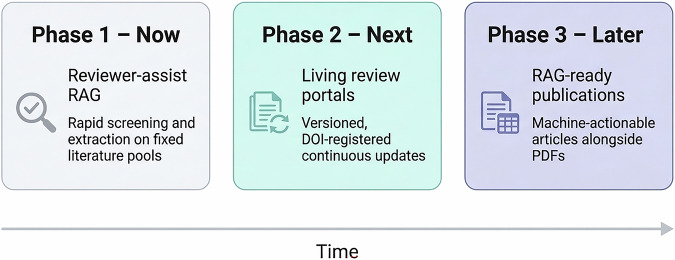
Adoption roadmap for Retrieval-Augmented Generation (RAG)–based dynamic evidence synthesis. The figure illustrates three progressive phases of integration. *Phase 1 (Now)* depicts reviewer-assist use of RAG for rapid screening and data extraction within fixed literature pools. *Phase 2 (Next)* shows the emergence of publicly accessible, versioned review portals that support continuously updated evidence synthesis. *Phase 3 (Later)* envisions RAG-ready scholarly publishing, in which machine-actionable articles are released alongside traditional PDFs, enabling immediate, query-time synthesis across the literature.

The introduction of the RAG methodology creates a clear distinction between two paradigms of evidence synthesis. The traditional systematic review, while rigorous and tested, produces a static snapshot of evidence. The RAG-powered dynamic review, in contrast, has the potential to create a living, interactive knowledge system. RAG can be integrated into evidence synthesis at increasing levels of depth, from reviewer-assist tools to public living portals and fully machine-actionable publications. Each level targets different PRISMA phases, has distinct benefits, and requires specific safeguards (Fig. 2).

### 1) Reviewer-assist RAG (“RAG-on-a-pool”) — now

Researchers can apply RAG to any pre-retrieved article set, regardless of how the pool was assembled. Within this constrained corpus RAG supports screening, data extraction, and evidence synthesis, improving recall by capturing semantic equivalents across synonyms and variants, accelerating extraction, and producing citation-anchored answers that remain fully auditable. To safeguard quality, users should validate recall on a gold-standard subset, require “answer-from-context only” prompts, log passage-level citations, and set predefined decision thresholds such as sensitivity targets for screening.

### 2) Public-facing living reviews (“RAG-backed review portals”) — near term

RAG can also power public-facing living reviews that readers browse and interrogate with natural-language questions^61^. Such portals surface scope-bounded answers with explicit sources, versioning, and a changelog of newly ingested studies, enabling continuous post-publication updates and a transparent record of evidence changes. By collapsing the update cycle from years to days, they keep topic pages current while maintaining scholarly rigor. Quality is safeguarded by freezing a citable baseline version (e.g., v1.0), providing detailed update notes, auditing retrieval quality, and registering a DOI with archived snapshots for reproducibility.

### 3) RAG-ready publications (“machine-actionable articles”) — medium to long term

Journals could eventually publish, alongside the traditional PDF, query-ready artifacts containing structured section and paragraph identifiers, table and figure metadata, method and outcome schemas, and an open API or packaged JSON so that any RAG system can immediately ingest and answer queries^62,63^. Integrated at the authoring and publishing stages, this approach would make downstream synthesis virtually instantaneous, eliminating PDF scraping, reducing ambiguity, and ensuring passage-level provenance^64^. To guarantee reliability, it would require persistent paragraph IDs, standardized table schemas with variables and confidence intervals, licenses permitting machine use, tamper-evident hashes, full documentation of embedding and indexing procedures, and systematic bias and error reporting.

### Extension to meta-analysis

The RAG framework can be extended to full meta-analytic workflows when quantitative outcome data are available. In a meta-analytic context, RAG can retrieve the relevant study chunks containing numerical results (e.g., means, standard deviations, confidence intervals, event counts), which can then be passed to an integrated statistical engine to compute effect sizes, perform random- or fixed-effects pooling, quantify heterogeneity, and produce forest or funnel plots. This creates a reproducible pipeline in which retrieval, extraction, statistical synthesis, and natural-language interpretation are all tightly coupled. Early studies support the feasibility of this approach. It has been shown that LLM-assisted extraction can reliably identify quantitative parameters from published articles, with high accuracy when statistical information is clearly reported, suggesting that RAG-grounded extraction could serve as a structured input layer for subsequent meta-analysis^65^. Likewise, AI-assisted systematic review pipelines can already automate search, screening, and structured data extraction, indicating that quantitative synthesis is a natural next step for AI-supported evidence workflows^44^. However, significant challenges remain—particularly automated extraction from tables and figures, managing ambiguous or inconsistently reported metrics, enforcing transparent provenance for each effect-size computation, and maintaining reproducible code signatures for regulatory or methodological auditing. Despite these limitations, integrating RAG with statistical computing environments represents a promising direction for creating continuously updated meta-analytic “living quantifications” that accompany narrative dynamic reviews.

## Implications for evidence-based dental practice and research

**Fig. 3:**
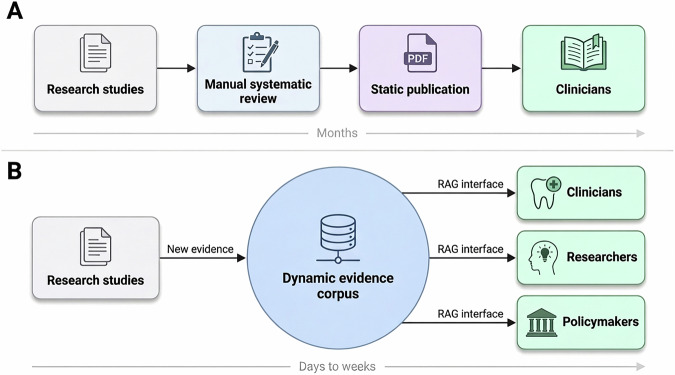
From static to dynamic evidence synthesis. **A** In the traditional evidence ecosystem, research studies are synthesized through manual systematic reviews and disseminated as static publications, resulting in substantial time delays before evidence reaches clinical practice. **B** In a dynamic evidence ecosystem, newly published studies are continuously incorporated into a dynamic evidence corpus. Clinicians, researchers, and policymakers access this corpus through a Retrieval-Augmented Generation (RAG) interface, enabling on-demand, citation-grounded evidence synthesis within days to weeks rather than months. This model supports more timely, transparent, and context-specific access to evidence across stakeholder groups.

Adopting RAG-powered dynamic reviews will reshape clinical decision-making, research workflows, and scholarly publishing (Table 1, Fig. 3).

**Table 1:** Compares four models of evidence synthesis: the traditional systematic review and three progressively more integrated RAG-based approaches—Reviewer-Assist RAG, Living Review Portals, and Machine-Actionable Publications.

Attribute	Traditional systematic review	Reviewer-assist RAG	Living review portal	Machine-actionable publication
Timeliness	Static; quickly becomes outdated.	Faster review on static pools; turnaround in days.	Continuously updated post-publication.	Real-time synthesis enabled at publication.
Search strategy	Manual, keyword-based; prone to missing synonyms and variations.	Semantic + keyword hybrid retrieval within preselected corpora.	User query triggers real-time hybrid retrieval.	Optimized semantic indexing at paragraph and schema level.
Scalability	Low; constrained by manual labor and time.	Moderate; fast review of pre-filtered pools.	High; handles evolving literature.	Very high; scalable ingestion and querying at field level.
Updateability	Requires full re-review to update.	Requires new pool; otherwise fast once retrieved.	Continuous ingestion and changelogs.	Continuous updates tied to structured, versioned content.
Transparency	Often opaque; traceability depends on authors.	Passage-level citations logged; context-constrained answers.	Citations, versioning, and evidence deltas visible.	Full provenance, schema-level metadata, and audit trails.
Resource intensity	High setup and high marginal costs.	High setup, low marginal cost per review.	Initial setup + editorial support; low marginal costs.	High setup; amortized over entire publishing pipeline.
User interaction	Passive; static documents (e.g., PDFs).	Analyst-driven; targeted QA workflows.	Interactive querying by readers.	Queries enabled from publication; machine-readable structure from day one.

Attributes span timeliness, search strategy, scalability, updateability, transparency, resource needs, and user interaction. The table reflects how RAG methodologies evolve from accelerating human workflows to enabling fully dynamic, query-ready evidence ecosystems.

For scholars, RAG compresses evidence retrieval from weeks to hours, allowing natural-language queries—such as *“For a patient with bruxism, what is the 5-year survival rate of monolithic zirconia crowns compared to lithium disilicate veneers?”*—to return immediate, citation-anchored summaries of the latest trials and meta-analyses. Accuracy can be strengthened by using LLMs pre-trained on scientific and medical literature. For researchers and educators, RAG systems accelerate literature mapping, hypothesis generation, and manuscript preparation, and will increasingly integrate multimodal data, from tables to radiographs, for richer synthesis.

The impact on publishing may be equally significant^66^. The current model—selling access to individual articles—could give way to licensing AI-ready data streams or offering premium AI-powered synthesis services. Peer review might shift from a single pre-publication check to continuous, post-deployment auditing of a RAG system’s accuracy, fairness, and bias^67^. To make machine-actionable publishing practical, stakeholders will need clear standards: structured article payloads with stable paragraph IDs and machine-readable tables, licenses that allow retrieval and indexing, APIs or bundled files with checksums, versioning and DOI conventions for dynamic updates, and a lightweight audit trail documenting indexing and embedding parameters. These measures would let any compliant RAG system ingest and answer queries without scraping, while preserving reproducibility.

Looking further ahead, emerging *agentic AI* systems—LLMs endowed with autonomous planning, memory, and the ability to trigger new retrieval or analytic steps—could extend RAG by not only answering queries but also initiating follow-up searches, designing mini-protocols, or coordinating multi-step evidence audits with minimal human prompting^52,68,69^.

This technological transformation also raises ethical concerns. Advanced LLMs and the infrastructure for robust RAG remain largely proprietary and costly, risking a new “evidence divide.” Well-resourced institutions may enjoy dynamic, living evidence, while individual practitioners, underserved clinics, and researchers in lower-income settings could be left with static, outdated reviews^70^. The ideal of democratized knowledge is therefore threatened by the economic models governing access. Global health agencies and professional societies must craft policies and non-profit initiatives to ensure equitable availability of these tools.

The RAG systems described here function as evidence-synthesis tools that retrieve, organize, and summarize published literature, rather than as software that generates patient-specific diagnostic or therapeutic recommendations. Under current regulatory frameworks—such as the EU Medical Device Regulation (MDR) and the FDA’s guidance on Clinical Decision Support—software that merely processes or contextualizes scientific literature, without analyzing individual patient data or directing clinical actions, is not classified as a medical device^71^. However, if future implementations of RAG were incorporated into clinical decision-support systems that directly influence patient management, they would fall under the category of Software as a Medical Device (SaMD) and require appropriate validation, performance monitoring, and regulatory oversight^72^. This regulatory boundary is not fixed and will likely evolve as AI-based evidence tools become more tightly integrated into clinical decision-making workflows. These distinctions are important to ensure safe integration of AI-enhanced evidence tools into dental practice.

## Conclusion: elevating human expertise in an AI-augmented future

RAG-powered dynamic reviews are not designed to replace human experts; rather, their limitations reinforce the continued necessity of expert judgment. Their primary role is to automate the most laborious and mechanical components of evidence synthesis—searching, screening, and aggregating large volumes of data—thereby freeing clinicians and researchers to focus on tasks that remain distinctly human. The decisive test of RAG is therefore not fluency, but reliability: whether these systems can consistently retrieve, ground, and cite evidence in ways that earn the trust of dental scholars. While these advantages are conceptually compelling, their clinical and methodological value ultimately depends on rigorous empirical evaluation.

To realize this potential, robust methodological frameworks are required to embed RAG within established evidence-synthesis workflows. Clear standards for corpus curation, retrieval quality, transparency of outputs, and integration with systematic review processes are essential. Without such safeguards, RAG risks remaining a promising but underutilized technology rather than a practical instrument for evidence-based dentistry.

For dental practice in particular, the value of dynamic RAG-based reviews lies in their ability to support point-of-care decision making while preserving the transparency demanded by evidence-based practice. Rather than supplanting systematic reviews, this approach complements them by allowing clinicians to interrogate the underlying evidence directly, verify claims at the passage level, and adapt queries to patient-specific contexts. In this way, dynamic reviews may shorten the distance between emerging dental research and everyday clinical application, without compromising methodological rigor.

At the same time, RAG-powered dynamic reviews remain a methodological proposal rather than a fully validated replacement for established approaches. Prospective empirical studies are needed to benchmark their performance against conventional systematic reviews, to assess retrieval completeness and citation fidelity, and to evaluate their behavior in real-world clinical and research settings. Addressing these questions represents a critical next step in translating RAG from a promising framework into a mature component of evidence-based dentistry.

As AI systems increasingly handle the *what* of evidence, the role of human expertise will shift toward the *so what* and the *what now*: critical appraisal, contextual interpretation, ethical judgment, and patient-centered application. As agentic AI matures, such systems may evolve from passive assistants to more proactive collaborators, but always within frameworks that safeguard transparency, accountability, and clinical responsibility. The future of evidence-based dentistry is therefore not one of automation, but of augmented intelligence—where technology elevates, rather than replaces, human expertise.

## Data Availability

No data were generated for this article.
